# Effective chemoimmunotherapy by co-delivery of doxorubicin and immune adjuvants in biodegradable nanoparticles

**DOI:** 10.7150/thno.34429

**Published:** 2019-08-21

**Authors:** Candido G. Da Silva, Marcel G.M. Camps, Tracy M.W.Y. Li, Luana Zerrillo, Clemens W. Löwik, Ferry Ossendorp, Luis J. Cruz

**Affiliations:** 1 Department of Radiology, Leiden University Medical Center (LUMC);; 2 Department of Immunohematology and Blood Transfusion, LUMC, Leiden, The Netherlands;; 3 Department of Radiology, Erasmus MC, Rotterdam, The Netherlands.

**Keywords:** chemoimmunotherapy, immune modulation, immune adjuvants, multi-drug nanoparticle, theragnostic.

## Abstract

Chemoimmunotherapy is an emerging combinatorial modality for the treatment of cancers resistant to common first-line therapies, such as chemotherapy and checkpoint blockade immunotherapy. We used biodegradable nanoparticles as delivery vehicles for local, slow and sustained release of doxorubicin, two immune adjuvants and one chemokine for the treatment of resistant solid tumors.

**Methods:** Bio-compatible poly(lactic-co-glycolic acid)-PEG nanoparticles were synthesized in an oil/water emulsion, using a solvent evaporation-extraction method. The nanoparticles were loaded with a NIR-dye for theranostic purposes, doxorubicin cytostatic agent, poly (I:C) and R848 immune adjuvants and CCL20 chemokine. After physicochemical and *in vitro* characterization the nanoparticles therapeutic efficacy were carried-out on established, highly aggressive and treatment resistant TC-1 lung carcinoma and MC-38 colon adenocarcinoma models *in vivo*.

**Results:** The yielded nanoparticles average size was 180 nm and -14 mV surface charge. The combined treatment with all compounds was significantly superior than separate compounds and the compounds nanoparticle encapsulation was required for effective tumor control *in vivo*. The mechanistic studies confirmed strong induction of circulating cancer specific T cells upon combined treatment in blood. Analysis of the tumor microenvironment revealed a significant increase of infiltrating leukocytes upon treatment.

**Conclusion:** The multi-drug loaded nanoparticles mediated delivery of chemoimmunotherapy exhibited excellent therapeutic efficacy gain on two treatment resistant cancer models and is a potent candidate strategy to improve cancer therapy of solid tumors resistant to first-line therapies.

## Introduction

Triggering antitumor immunity through chemotherapy, immunotherapy, or combinations thereof is an emerging strategy to treat solid tumors 1. Besides killing cancer cells directly, some chemotherapies can alter the tumor microenvironment and enhance immune responses 2,3. For example, the anthracycline doxorubicin (dox) has been described to induce type I interferons (IFNs), T cell homing through induction of the chemokine CXCL10, expose calreticulin on dying cells, and other effects 2,4. However, dox monotherapy is often insufficient to clear established solid tumors, eliciting the need for combinatorial modalities.

Immunotherapy based on immune adjuvants such as cytokines, checkpoint blocking antibodies, Toll-like receptor (TLR) agonists and other compounds, are gaining attention as a strategy to enhance anticancer immune responses 5-9. TLR agonists trigger broad inflammatory responses, elicit rapid innate immunity, promote the activity of leukocytes, and facilitate the progression from innate to adaptive immune responses 10. Moreover, TLRs facilitate the immune system by providing context, allow the immune system response to skew on the type that is necessary and finetune the most efficient method to eradicate the threat to the host. Numerous TLR agonists have been studied as cancer therapies (or part of combination therapies) in clinical trials. Intriguingly, several agonists have demonstrated antitumor effects, whereas others appear to promote tumor growth or metastasis 11. In humans, activation of the endosomal TLR3, TLR7, TLR8 and TLR9 typically enhances antitumor outcomes. For example, the TLR3 agonist Poly(I:C; pIC) has been reported to have potent antitumor effects on lung and liver cancers, and the dual TLR7/8 activator Resiquimod (R848) has been reported in several clinical trials to induce tumor regression in patients with advanced leukemia and skin cancers 11,12. Moreover, R848 has been reported to reverse effector T cell senescence 13. Interestingly, the combination therapy of pIC and R848 appears to be synergistic *in vitro*, but this effect has not yet been demonstrated in clinical trials 14.

To date, most clinical trials on TLR agonists involved the systemic administration, which led to deleterious adverse effects, including cytokine release syndrome, which can rapidly become fatal. Thus, the anticancer efficacy of TLR agonists is limited by systemic treatment. Accordingly, TLR agonists are being actively explored within combination therapies administered intratumorally. Chemokines are specific immune adjuvants that can induce chemotaxis of immune cells to the tumor, thereby making tumors more visible to immune cells. Similarly to TLR agonists, some chemokines may exert anticancer effects, whereas others may enhance cancer progression depending on the cancer type, the tumor microenvironment phenotype, and the cancer stage 15. One chemokine that can drive immune cells towards the tumor is the Macrophage Inflammatory Protein-3 alpha (MIP3α; CCL20) which attracts cells expressing CCR6/CD196 such as (memory) T cells, natural killer cells and immature dendritic cells (DCs), all of which can mediate tumor regressions 16-19. Furthermore, MIP3α has also been described to directly repress the proliferation of myeloid progenitors 20.

Successful therapeutic responses are commonly observed when the effective dose of a drug is maintained at the target site for a specific duration. However, drugs that are administered systemically can generate numerous off-target effects that compromises the therapy efficacy. In response, either the dose is adjusted or the treatment is stopped, both of which can be problematic for the survival of the patient. Therefore, for certain anticancer drugs, local administration may prove more effective than systemic administration 5. However, one disadvantage of local treatment is rapid diffusion, which limits efficacy. Therefore, an attractive route of administration would be one that is local, to avoid off-target effects, but in which the drug is released slowly for a sustained period, to maximize efficacy. This approach entails the use of drug delivery vehicles such as liposomes, metallic nanoparticles (NPs) or biodegradable poly(lactic-co-glycolic acid; PLGA) polymers 21,22. Indeed, delivery of cancer therapeutics with such vehicles is rapidly gaining recognition for its advantages. For instance, over the past several years, the FDA approved nano-vehicle formulations of previously developed chemotherapeutics: Doxil®, Abraxane®, and Onivyde® for dox, paclitaxel, and irinotecan, respectively. Interest in drug delivery vehicles is also reflected by the large number (>200) of clinical trials currently underway in which chemotherapeutics are being compared to their respective soluble and delivered forms 23-25.

Herein, we report the assembly and *in vitro* functional characterization and loading of PLGA NPs with dox, pIC, R848 and MIP3α, and subsequent *in vivo* evaluation of the loaded NPs as a cancer therapy. We assessed the activity of our drug-loaded NPs in two aggressive and treatment resistant murine models of cancer: TC-1 lung carcinoma and MC-38 colon adenocarcinoma. We provide evidence of enhanced potential of chemotherapy and immunotherapy. Finally, we investigated the *in vivo* efficacy of the NP delivered drugs against the corresponding free drugs and analyzed the tumor microenvironment. To the best of our knowledge, this is the first published study to combine NP mediated delivery of a chemotherapeutic agent, two distinct TLR agonists and a chemokine into a single theranostic modality.

## Materials and methods

### Materials and reagents

PLGA polymer (lactide/glycolide molar ratio of 48:52 to 52:48) was purchased from Boehringer Ingelheim (Ingelheim am Rhein, Germany). Solvents for synthesizing the PLGA NPs including dichloromethane (DCM; CAS 75-09-2 CH2CL2 MW 84.93) and polyvinyl alcohol (PVA; CAS 9002-89-5) were purchased from Sigma-Aldrich (Zwijndrecht, The Netherlands). Chloroform (CHCL3 MW 119.38 g/mol) was purchased from Merck (Darmstadt, Germany). Lipid-PEG 2000 (1,2-Distearoyl-sn-Glycero-3-Phosphoethanolamine-N-[Methoxy(Polyethylene glycol)-2000]; powder MW 2805.54) was purchased from Avanti Polar Lipids (AL, USA). The near infrared (NIR) dye (IR-780 Iodide; CAS 207399-07-3) was purchased from Sigma-Aldrich; R848 from Alexis Biochemicals (Paris, France); poly(inosinic:cytidylic acid; CAS 42424-50-0 P0913) from Sigma-Aldrich; MIP3α from R&D Systems (MN, USA) and doxorubicin HCL powder from Actavis (Munich, Germany).

### Synthesis of PLGA NPs

The NPs were synthesized in an oil/water emulsion, using a solvent evaporation-extraction method. Briefly, 200 mg of PLGA was dissolved in 6 mL of DCM containing 1 mg of NIR dye. Depending on the NP, the following was added: 40 mg of dox, 8 mg of pIC and/or 4 mg of R848 and/or 250 µg of MIP3α. Next, the solution containing the NP constituents was added dropwise to 40 mL of aqueous 2.5% (w/v) PVA and emulsified for 120 s using a sonicator (250 watt; Sonifier 250; Branson, Danbury, USA). Next, the previously described solution was transferred to a new vial that contained an air-dried solution of 40 mg of Lipid-PEG 2000 dissolved in 0.4 mL of chloroform and homogenized for 60 s by sonication. Following overnight evaporation of the solvent at 4 °C, the NPs were collected by ultracentrifugation (12,800 rpm for 30 minutes) at 4 °C, washed four times with distillated water, and lyophilized for 3 days. The concentration of each encapsulated constituent (dox, pIC, R848 and MIP3α) was determined by distinct methods, as described elsewhere 26. In brief, the concentration of the TLR agonists (pIC and R848) were determined by reverse phase high-performance liquid chromatography (RP-HPLC) at room temperature using a Shimadzu system (Shimadzu Corporation, Kyoto, Japan) equipped with a RP-C18 symmetry column (250 mm x 4.6 mm). The flow rate was fixed at 1 mL/min and detection was obtained by UV detection at 254 nm. A linear gradient of 0% to 100% of acetonitrile (0.036% TFA) in water containing 0.045% TFA was used for the separation of pIC and R848. The peak of R848 was well separated from that of the pIC in the established chromatographic condition. The retention times of the pIC and R848 were approximately 19 and 26 min, respectively. The regression analysis was constructed by plotting the peak-area ratio of R848 or pIC versus concentration (µg/mL). The calibration curves were linear within the range of 1 µg/mL to 10 µg/mL for R848 and 1 µg/mL to 150 µg/mL for pIC. The correlation coefficient (R2) was always greater than 0.99, indicating a good linearity. The concentration of pIC and R848 was calculated by interpolation into the standard curves as described previously. The concentration of MIP3α was determined by RP8-HPLC at room temperature using a Shimadzu system (Shimadzu Corporation) equipped with a RP-C8 symmetry column (150 mm x 4.6 mm). The flow rate was fixed at 0.8 mL/min and detection was obtained by UV detection at 220 nm. A linear gradient of 5% to 80% of acetonitrile (0.036% TFA) in water containing 0.045% TFA was used. The concentration of the NIR dye was measured at 800 nm relative to a standard curve using an Odyssey scanning (Li-Cor) as per described previously 27. The dox concentration was determined by SpectraMax® iD3 multi-mode microplate readers via fluorescence with an excitation peak at 488 nm and emission peak at 530 nm. The loading capacity was calculated as follows: Percentage loading capacity = [entrapped drug /NP yield weight] * 100

### Physicochemical properties of the NPs

The NPs were characterized for average size, polydispersity index and surface charge (zeta-potential) by dynamic light scattering. Briefly, 50 µg of NP sample in 1 mL of ultrapure MilliQ H_2_O were measured for size using a Zetasizer (Nano ZS, Malvern Ltd., UK) and a similar sample was analyzed for surface charge by laser Doppler electrophoresis on the same device.

### Particles surface and morphology

To visualize the structure of the NPs, transmission electron microscopy (TEM) was used. Briefly, a formvar support film attached to a copper grid (100 mesh) was coated with carbon and hydrophilized by glow-discharging for 30 s with a current of 25 mA. A droplet of 3 µL of the NPs solution was applied to the grid and then stained for 1 min in distilled water containing 2.3% uranyl acetate. Next, the grid was air-dried and imaged in a Tecnai 12 Biotwin transmission electron microscope (FEI, The Netherlands), equipped with a LaB6 filament operated at 120 keV. The sample was imaged 3 µm under focus with binning 2 on a 4kx4k Eagle CCD camera with a magnification of 18,500x.

Atomic force microscopy (AFM) was employed to study the surface morphology and size of NPs. Briefly, a drop of diluted and dispersed NPs suspension was placed on a clean glass surface glued to the AFM stub. The dried NPs were then visualized with AFM (JPK Nano Wizard 3) in AC mode (tapping mode), using OMCL-AC160TS silicon probes (Olympus), with nominal resonance frequency of 300 kHz and nominal spring constant of 26N/m. The images were analyzed using Gwyddion SPM Software (Czech Metrology Institute, Czech Republic). The 2D visualization was performed with JPK Data Processing Software (JPK Instruments, Germany) and the images were converted to 3D using Gwyddion v. 2.52 (open source SPM data analysis software).

### Stability study and release kinetics of the NPs

For the NP stability study a total of 10 mg of each described NP was carefully dissolved in 2 mL of PBS and kept at room temperature and at constant rotating velocity. At the designated time points a 50 µL sample was taken from the supernatant and measured by dynamic light scattering as per described above. For the NP release kinetics study, 1 mL (10 mg/mL) of the NP containing all drugs was pipetted into a dialysis bag (MWCO 1000), which was immersed into a tube containing 30 mL of PBS (pH 7.4). The tubes were placed on a shaking bed at 100 rpm and 37 °C. At the described time points, 30 mL of the release medium was collected and replenished with 30 mL of fresh PBS. The collected sample was concentrated by lyophilization in order to determine the content released for all components. The dox, NIR dye, TLR agonists R848 and pIC concentration were determined as per described above.

### Cell lines

The murine tumor cell line TC-1 (a kind gift from T.C. Wu, Johns Hopkins University, Baltimore, MD, USA) was generated by retroviral transduction of lung fibroblasts of C57BL/6 origin, to express the HPV16 E6 and E7 genes and the activated human c-Ha-ras oncogene 28. The C57BL/6 MC-38 colon adenocarcinoma cell line was kindly provided by Mario Colombo. The D1 cell line is an immature splenic DC line derived from B6 mice which harbors most of the typical characteristics of that of bone marrow derived DCs 29. The TC-1 cell line was cultured in DMEM medium (BioWhittaker, Verviers, Belgium) supplemented with 8% heat-inactivated fetal calf serum (FCS; Greiner bio-one, Alphen a/d Rijn, The Netherlands), penicillin (50 μg/mL; Gibco, Paisley, Scotland), streptomycin (50 μg/mL; Gibco), L-glutamine (2 mM; Gibco) and β-mercaptoethanol (20 μM; Sigma, Saint Louis, USA). In addition, the TC-1 cells were co-cultured with the corresponding selective agent Geneticin (G418; 400 μg/mL). The BALB/macrophage cell line RAW264.7 and the MC-38 cell line were cultured identically to the TC-1 cell line except that IMDM medium was used and no selection agent was applied. The D1 cell line was cultured as described previously 30. All the above described cell lines were incubated at 37º C in 5% CO_2_ and 100% humidity. Furthermore, the cell lines were confirmed to be free of mycoplasma and were regularly tested for eighteen common rodent viruses by PCR analysis.

### Mice strains

C57BL/6 (H-2^b^ haplotype) mice were purchased from Envigo (Horst, The Netherlands). They were all female and ranged in age from 8 to 12 weeks. The mice were housed at the animal facility of Leiden University Medical Center under specific pathogen free conditions. All animal experiments were approved by the Dutch Central Committee on Animal Experimentation and were strictly conducted according to the Dutch animal welfare law.

### Intracellular uptake of NPs and immunostaining

Intracellular uptake of NPs was determined by incubating either 10 µg/mL or 20 µg/mL of NPs containing NIR dye (~ 800 nm; described above) with 1x10^4^ TC-1 or D1 cells for 1 hour, 2 hours or 4 hours. To remove unbound NPs from the cells and wells, the cells were harvested and moved to a new 96-well plate and washed several times. Then, the cells were placed in a black 96-well microplate (Greiner bio-one, Germany), fixed with 1% paraformaldehyde (PFA) and stained with To-pro 3 iodide (642/661 ~700 nm; Invitrogen; Eugene, USA) to enable cell count. Finally, the NIR dye signal in each cell line was scanned using an Odyssey scanner infrared imaging system (LI-COR). Immunostaining detected by fluorescence microscopy was determined by incubating 20 µg/mL of NPs containing NIR dye with TC-1 or D1 cells in the chambers of a glass culture slide (FALCON, NY, USA) for 48 hours. After washing, and fixating the cells with 4% PFA, the cells were stained with anti-CD44-PE (clone GL1, eBioscience) for membrane visualization, washed again with PBS and finally, mounted with VectaShield antifade mounting medium with DAPI to stain nuclei (Vector Laboratories, CA, USA). Digital images were acquired using a Leica DM6B microscope.

### Activation and maturation of DCs

DC activation and maturation were assessed based on upregulation of CD86 on the D1 cells and production of IL-12 in the supernatant. Briefly, a solution of pIC and an equivalent concentration of pIC encapsulated in NPs, that also contained R848 and MIP3α, were separately prepared according to annotated concentrations (see corresponding figure legends). The solutions were then distributed into 96-well plates and sequentially diluted, after which 5x10^4^ D1 cells were added to each well and allowed to incubate for 48 hours at 37º C in 5% CO_2_ and 100% humidity. The supernatant was then harvested and analyzed with an ELISA (described below). The cells were used to analyze the CD86 expression with anti-CD86-APC (clone GL1, eBioscience) on an LSR-II laser flow cytometer controlled by CELLQuest software v. 3.0 (Becton Dickinson, Franklin Lakes, USA) and analyzed with FlowJo LLC v. 10 software (Tree Star, USA). The interleukin IL-12 was detected using a standard sandwich ELISA with bottom polystyrene ELISA plates (Corning, Kennebunk, USA). Purified anti-mouse IL-12/IL-23 p40 (clone C15.6, Biolegend) and biotin-labelled anti-mouse IL-12/IL-23 p40 antibodies (clone C17.8, Biolegend) were used. Streptavidin-horse radish peroxidase (1 μg/mL; Biolegend) and 3,3′,5,5′-tetramethyl benzidine (TMB; Sigma-Aldrich) was used to generate the detection signal. Finally, the plates were read at 450 nm using a Bio-rad 680 microplate reader (Bio-rad Laboratories).

### Cytotoxicity of empty and dox-loaded NPs

The toxicity of empty NPs to DCs was determined by incubating DCs (5x10^4^) with increasing concentrations of empty NPs for 48 hours, and then measuring cell viability. The cytotoxic compound dimethyl sulfoxide (DMSO; CAS 67-68-5; Honeywell, MI, USA) 25% (v/v) in medium was included as a positive control (100 percent cell death). To measure viability, the cells were stained with 7-AAD (Invitrogen) using standard protocols and then subjected to flow cytometry measurements on an LSR-II laser flow cytometer controlled by CELLQuest software v. 3.0 (Becton Dickinson). The cell toxicity of the dox-loaded NPs and controls was determined by using the CellTiter 96 AQueous one solution cell proliferation assay (MTS; Promega, Madison, USA) performed per manufacturer's instructions. In brief, 5x10^3^ cells per well were distributed into a 96-wells plate and treated with indicated concentrations of compounds at 37º C in 5% CO_2_ and 100% humidity. After 72 hours, cells were incubated with MTS solution before measuring absorbance at 490 nm using a Bio-rad 680 microplate reader (Bio-rad Laboratories).

### Transwell chemotaxis assay

A solution of NP(pIC+R848+MIP3α) in full medium was prepared at an equivalent MIP3α concentration of 1 µg/mL. Separately, a solution of free MIP3α at a matching concentration of 1 µg/mL, and a positive control solution of free MIP3α at 10 µg/mL, were prepared and distributed into the wells of a Transwell permeable 24-well plate (12x6.5 mm inserts; 8.0 µm PET membrane (Costar Corning, Kennebunk, USA). After 24 hours of incubation at 37 ºC, to allow sufficient MIP3α to be released from the NPs, the insert was pre-warmed with warm complete culture medium and the lower chamber solution was carefully re-suspended to homogenize MIP3α into the solution. Next, 1x10^5^ RAW264.7 cells were carefully added to each upper chamber insert and allowed to migrate for 24 hours. Next, the cells were fixed with 4% PFA, washed and stained with a crystal violet solution, after which several digital pictures of each insert were acquired with a reverse microscope. Cell migration was quantified using Image J software v. 1.5. The migration index was calculated by dividing the area (%) of migrated cells by the area (%) of migrated cells induced by the positive control.

### Tumor challenge with NP-delivered combination therapy

Mice were inoculated with 1x10^5^ TC-1 or 4x10^5^ MC-38 cells in 0.2 mL PBS in the right flank. When the tumors became established at day 8 after tumor inoculation, each mouse received a 30 μL intratumoral injection of NPs dissolved in PBS and this was repeated every other day (four injections in total), unless otherwise specified. The control (untreated) group received an intratumoral injection of 30 μL PBS every other day (four injections in total), unless otherwise specified. Each intratumoral treatment administration contained, in total: 1.5 mg/Kg (30 µg) of dox, 1.2 mg/Kg (24 µg) of pIC, 375 μg/Kg (7.5 µg) of R848, and 75 µg/Kg (1.5 µg) of MIP3α in NP stock concentration of ca. 50 mg/mL. Concentrations were matched for the groups treated with free therapies. The limiting concentration of NPs for the experiments (see figure legends) was the MTD of dox: 6 mg/Kg (4x 1.5 mg/Kg) 31. For the reduced dose experiment, the cumulative dose was 3 mg/Kg. For the dox and immune adjuvants combined experiments, pIC, R848 and MIP3α content was matched among groups on dox or on pIC, R848 or MIP3α content. Tumor dimensions were measured every other day with a standard caliper and the volume was calculated by multiplying the tumor diameters in all three dimensions. The maximal allowed tumor volume was 2,000 mm3; after this point, mice were sacrificed, which formed the basis for the Kaplan-Meier survival curves.

### Blood analysis

The presence of antigen-specific T cells in the blood of each mouse was determined by collecting 50 µL of blood via a puncture of the caudal vein at day 8 and day 16 after the first treatment. After removal of red blood cells by lysis, the cells were stained with anti-CD8α-PE (clone 53-6.7, eBioscience) and anti-CD3-eFluor 450 (clone 17A2, eBioscience). For mice bearing TC-1 tumors, the APC labeled HPV16 E7_49-57_ (RAHYNIVTF) MHC class I (H-2D^b^) tetramer was added to the staining mix. After thorough washing, the cells were subjected to flow cytometry measurements on an LSR-II laser flow cytometer controlled by CELLQuest software v. 3.0 (Becton Dickinson) and the data analyzed with FlowJo LLC v. 10 software (Tree Star).

### Tumor microenvironment and spleen analysis

The tumor microenvironment and the spleens of mice were analyzed ex vivo by sacrificing the mice and resecting the tumors and the spleens at day 18 after tumor inoculation (after a single treatment at day 8). From the six mice per group, only four mice were selected for analysis based on their similar tumor size. The resected tumors were then mechanically broken up into small pieces of ~2-3 mm in diameter (with sterile tweezers and scissors) and incubated with Liberase TL (Roche, Mannheim, Germany) in serum-free IMDM medium for 15 minutes at 37 ºC. Single cell suspensions of the tumors and the spleens were acquired by gently grinding the tumor fragments and the spleens through a 70 µm cell strainer (Falcon, NY, USA) each in separate 50 mL tubes. The red blood cells from the spleens where removed by lysis. Each tube containing the single cells were then equally divided to be stained with two distinct antibody panels. One panel contained the viability dye 7-AAD (Invitrogen) and the following antibodies against cell surface markers: anti-CD45.2-APC eFluor 780 (clone 104, eBioscience); anti-CD3-eFluor 450 (clone 17A2, eBioscience); anti-CD4-Brilliant Violet 605 (clone RM4-5, Biologend), and anti-CD8α-APC-R700 (clone 53-6.7, BD Bioscience). The other panel contained the viability dye 7-AAD (Invitrogen) and the following antibodies against cell surface markers: anti-CD45.2-FITC (clone 104, BD Bioscience); anti-CD11b-eFluor 450 (clone M1/70, eBioscience); anti-F4/80-PE (clone BM8, eBioscience); anti-Ly6G-AlexaFluor 700 (clone 1A8, Biolegend); anti-Ly6C-Brillian Violet 605 (clone HK1.4, Biolegend), and anti-CD11c-APC-eFluor 780 (clone N418, eBioscience). After thorough washing, the cells were subjected to flow cytometry measurements on an LSR-II laser flow cytometer controlled by CELLQuest software v. 3.0 (Becton Dickinson) and the data analyzed with FlowJo LLC v. 10 software (Tree Star). The gating strategy is depicted in Figure S1.

### Data and statistical analysis

Statistical analysis was performed using GraphPad Prism v. 7.0 software (GraphPad Software, La Jolla, USA). Data are represented as mean values ± SD unless stated otherwise. Tumor volumes, blood tetramer and tumor and spleen cell analysis results were compared on a fixed day between mouse groups and statistical significance was determined by using an unpaired, non-parametric, two-tailed Mann-Whitney U test. Survival curves were compared using the Gehan-Breslow-Wilcoxon test unless stated otherwise. Statistical differences were considered significant at p < 0.05 and presented as: * p < 0.05, ** p < 0.01, *** p < 0.001.

## Results

### Physicochemical properties and *in vitro* characterization of the NPs

**Figure 1:**
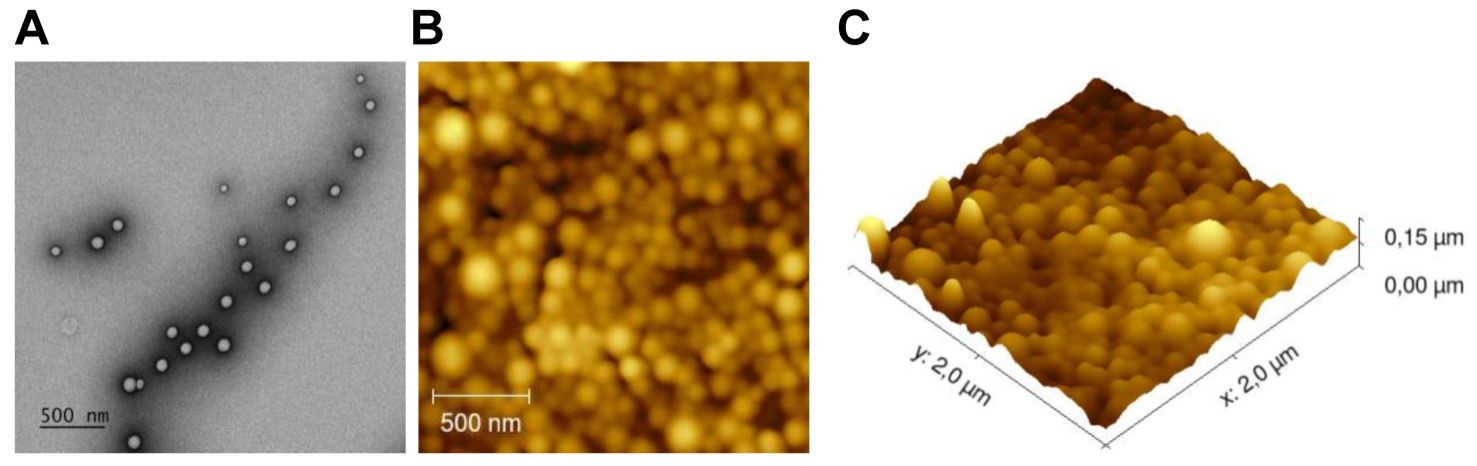
** NPs surface and morphology. (A)** Representative morphology image of NP(empty) obtained by TEM. **(B)** AFM 2D image. **(C)** AFM 3D image.

We loaded NPs with dox and/or different immune adjuvants and then studied their therapeutic potential (Table 1). The tumor immunity of the monotherapy NPs containing only immune adjuvants were studied separately (manuscript submitted). Due to the limited *in vivo* detection capability of the fluorescent anthracycline doxorubicin, we loaded a NIR dye in each batch of NPs to enable *in vivo* theranostic analysis and the NPs were functionalized with surface PEGylation (PEG). The NPs were first characterized to ascertain their size and surface charge (Table 1 and Figure S2). The average size was approximately 180 nm and differed depending on the cargo. The average ζ potential was slightly negative: ca. -14 mV. The NPs were stable in PBS for at least 8 weeks (Figure S3). TEM and AFM analysis revealed that the NPs were all spherical with a smooth surface and uniform sizes (Figure 1).

### Drug release kinetics

**Figure 2:**
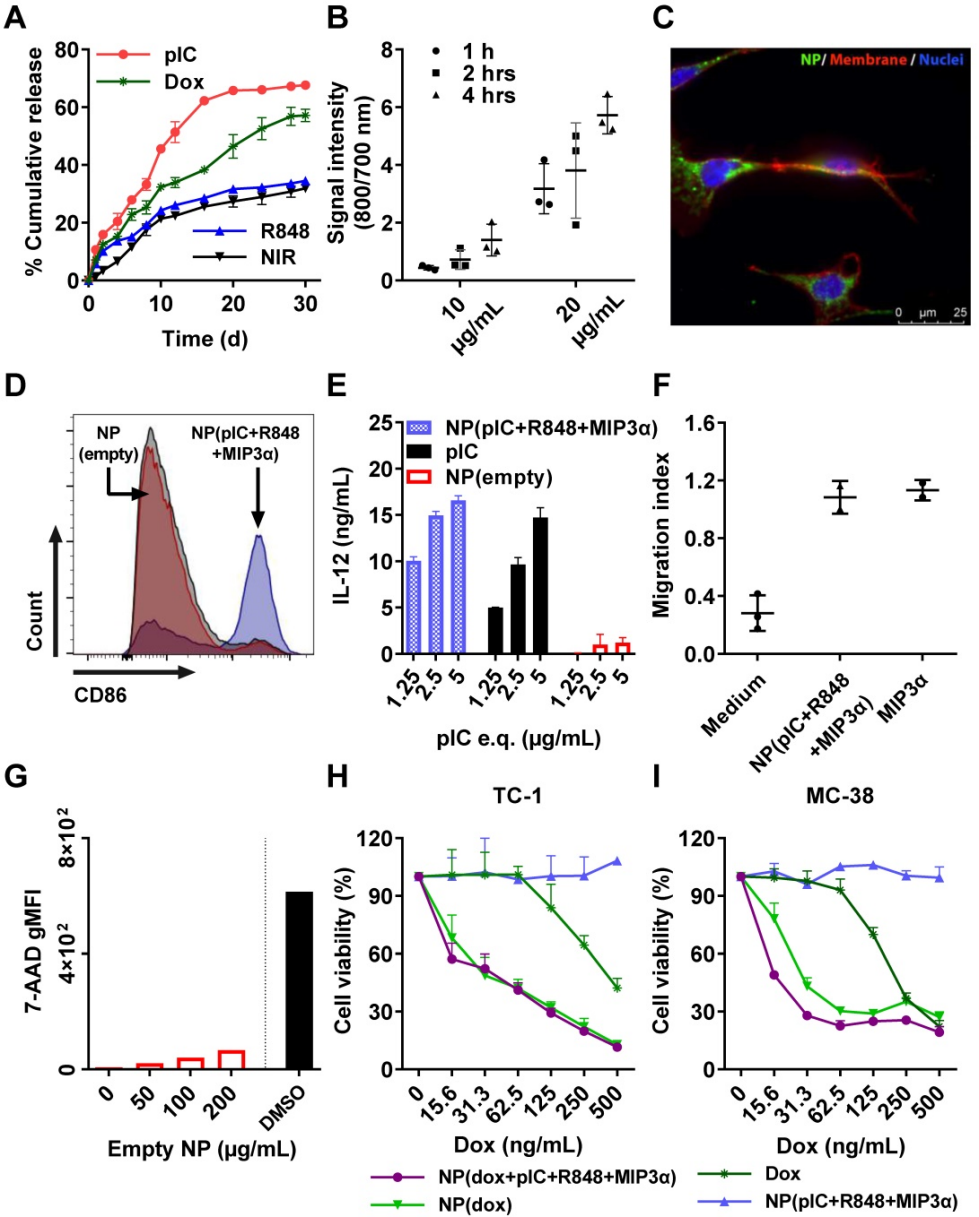
***In vitro* cumulative release kinetics, cellular uptake, DC activation, and cytotoxicity of the empty and drug-loaded NPs**. **(A)** NP release kinetics of encapsulated drugs simulated at 37°C in PBS and kept in a thermo-shaker at a constant shaking velocity. n = 3 from one representative experiment. **(B)** Uptake of NPs containing NIR dye (800 nm) by TC-1 cells (To-pro 3 iodide; 700 nm) over the times indicated. n = 3 from one representative experiment. **(C)** Uptake of NPs by TC-1 cells after 2 hours of incubation, shown by fluorescence microscopy. Red: cell membrane; purple: cell nucleus; green: NIR dye. **(D)** Activation of DCs measured by CD86 expression upon 48 hours incubation with NP(pIC+R848+MIP3α). NP(empty) and isotype controls are shown in red and grey, respectively. The cells were pooled from n = 3 from each condition, one representative out of three independent experiments. **(E)** Activation of DCs measured by the secretion of IL-12p40 upon 48 hours incubation with NP(pIC+R848+MIP3α). NP(empty) and pIC controls are shown in red and black, respectively. n = 3 from one representative out of three independent experiments.** (F)** Migration assessment using Boyden chamber assay. After 24 hours of pre-incubation of the lower chamber with either MIP3α (in solution) or NP(pIC+R848+MIP3α), RAW264.7 cells were added to the upper chamber and allowed to migrate for 24 hours. Medium was used as a negative control. n = 3 from one representative out of two independent experiments. **(G)** Cytotoxicity measurement of empty NPs on DCs incubated with increasing concentrations for 48 hours. The cytotoxic compound DMSO (black bar) was used as a positive control (100 percent of cell death). (H+I) Cell viability assessed by MTS cell proliferation assay upon 72 hours incubation with indicated compounds on TC-1 **(H)** or MC-38 **(I)** cells. n = 3 from one representative out of four independent experiments. All data are presented as mean ± SD.

We measured the drug release kinetics of the NPs dissolved in PBS and kept at 37°C in a thermo-shaker at a constant shaking velocity. The NPs exhibited a sustained release profile with different release kinetics for each drug (Figure 2A). After 12 days, approximately 50% of pIC was released, 35% of dox, 25% of R848 and the NIR dye, respectively. MIP3α release could not be determined because it was below the detection limit. The profile release of pIC was the most rapid compared to the other drugs due to its high hydrophilicity property. The other encapsulated compounds show a typical drug profile release from the PLGA (lactide/glycolide molar ratio of 50:50) standard polymer. These results suggest that the NPs release drugs in a slow, sustained manner.

### Cellular uptake of the NPs

Since dox, pIC and R848 all exert their biological effects intracellularly (unlike MIP3α), we sought to assess the uptake of drug-loaded NPs by cells. To this end, NPs containing NIR dye (at 10 µg/mL and at 20 µg/mL) were incubated with TC-1 cells for 1 hour, 2 hours and 4 hours (Figure 2B). At 10 µg/mL, the signal was detected after 2 hours and 4 hours of incubation, but not after 1 hour. At 20 µg/mL, the signal was detected at all three time points, and it increased with increasing incubation time. To determine whether the signal was originating from inside the cells, the NPs were incubated with TC-1 cancer cells again for 2 hours at 20 µg/mL and observed under fluorescence microscopy (Figure 2C). The NIR signal (green) from the NPs was observed within cells, indicating that the NPs had released their content into the cells. Similar results were observed when these experiments were performed with DCs instead of TC-1 cells (data not shown).

### NPs enhance DC activation, IL-12 production, and induce chemotaxis

The ligands pIC and R848 are agonists for the endosomal TLR3 and TLR7/8, respectively, which are predominantly located inside cells. Activation of TLR3 or TLR7/8 can be detected by measuring the expression of CD86 in D1 DCs. For this purpose, NP(pIC+R848+MIP3α) was incubated at increasing concentrations with DCs for 48 hours. The loaded NPs caused a dose-dependent increase in CD86 expression, whereas empty NPs at equivalent concentrations did not (Figure 2D). Moreover, incubation with NP(pIC+R848+MIP3α) triggered IL-12 secretion by DCs, indicating that these cells had been activated and that the TLR agonists in the NPs had remained active (Figure 2E). To determine the activity of MIP3α after co-encapsulation in NPs, the chemotactic capacity of this chemokine was assessed by incubating NP(pIC+R848+MIP3α) with medium in the lower chamber of a transwell system (Figure 2F). MIP3α was observed to attract approximately three times the number of cells across the membrane compared to medium only, indicating that, like the TLR agonists, MIP3α also had remained active after co-encapsulation in the NPs.

### Cytotoxicity of empty and loaded NPs

We next sought to determine the cytotoxicity of the empty and loaded NPs (dox only, immune adjuvants only or combinations thereof). First, DCs were co-cultured *in vitro* with empty NPs for 48 hours at increasing NP concentrations, subsequently stained with the cell death marker 7-AAD, and finally, analyzed by flow cytometry (Figure 2G). The empty NPs did not induce any significant cytotoxicity, as measured by the low signal of 7-AAD relative to the signal of the DMSO control. Next, to ascertain the effects of loading dox into NPs on its chemotherapeutic activity, an MTS cytotoxicity assay was performed by treating TC-1, MC-38 cells and DCs with dox-loaded NPs (Figures 2H, 2I and S4A, respectively). In all cell lines, cytotoxicity was dose-dependent. For TC-1 and MC-38 the dox-loaded NPs provoked ten times the level of cell death as did the free dox. The LD50 of dox in MC-38 cells (ca. 200 ng/mL) was half of that of TC-1 cells (ca. 400 ng/mL). However, the NPs with immune adjuvants alone did not induce cell death in either cell line. In addition, we compared the effect of multi-drug encapsulation of NP(dox+pIC+R848+MIP3α) and of NP(pIC+R848+MIP3α) versus non-encapsulated (soluble) controls on cell viability (Figure S4). NP(pIC+R848+MIP3α) or the soluble controls did not affect cell viability. On the other hand, NP(dox+pIC+R848+MIP3α) was more efficient in killing cells than the soluble controls. Overall, these results indicate that empty NPs are non-cytotoxic to DCs and that NP-delivered dox shows greater cytotoxicity to two cancer cell lines than does free dox.

### Intratumoral co-delivery of dox with immune adjuvants boosts lymphocyte influx in the tumor microenvironment

**Figure 3:**
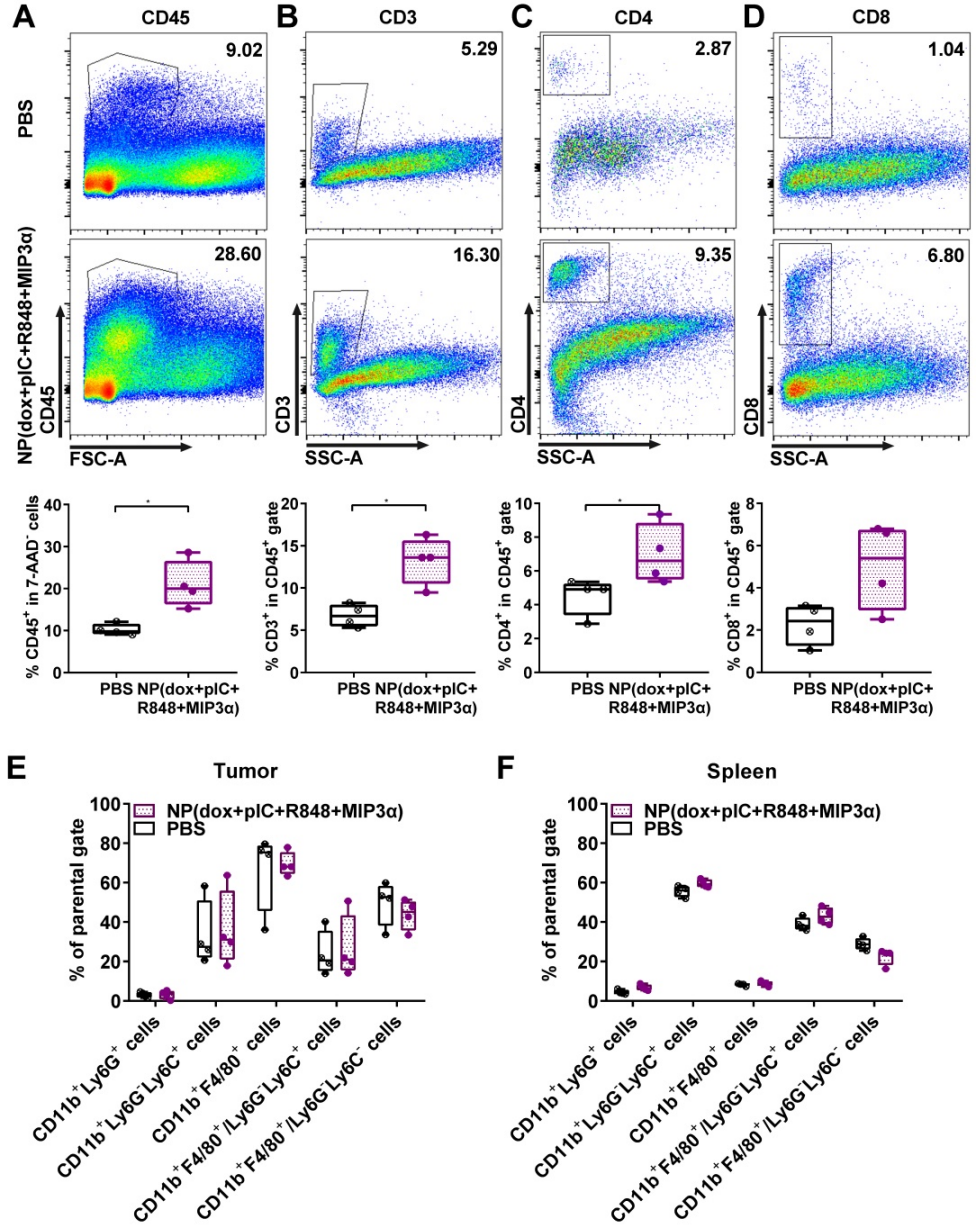
** Intratumoral co-delivery of dox with immune adjuvants boosts lymphocyte influx in the tumor microenvironment.** At day 8, mice with TC-1 tumors received a single intratumoral injection of either PBS (mock control) or NP(dox+pIC+R848+MIP3α). Ten days later, the tumors were resected and analyzed by flow cytometry: **(A)** Representative flow cytometry plot showing CD45.2 cells in a mock (PBS) or treated tumor. The box and whiskers plot depicts n = 4 from one representative out of two independent experiments (p=0.0286). **(B)** Representative flow cytometry plot showing CD3^+^ cells in a mock (PBS) or treated tumor. The box and whiskers plot depicts n = 4 from one representative out of two independent experiments (p=0.0286). **(C)** Representative flow cytometry plot showing CD4^+^ T cells in a mock (PBS) or treated tumor. The box and whiskers plot depicts n = 4 from one representative out of two independent experiments (p=0.0286). **(D)** Representative flow cytometry plot showing CD8^+^ T cells in a mock (PBS) or treated tumor. The box and whiskers plot depicts n = 4 from one representative out of two independent experiments (p=0.1143; n.s.). **(E)** Different cell types within the myeloid population analyzed in the tumor is depicted upon mock treated (PBS) tumors or treated tumors. n = 4 from one representative out of two independent experiments. **(F)** Different cell types within the myeloid population analyzed in the spleen is depicted upon mock treated (PBS) tumors or treated tumors. n = 4 from one representative out of two independent experiments. Statistics were calculated using a two-tailed Mann Whitney test. Statistical differences were considered significant at p < 0.05. * = p < 0.05; ** p = < 0.01; *** p < 0.001. Data plotted are presented as min to max.

To assess alterations in the tumor and spleen upon treatment, we analyzed the lymphoid and myeloid populations of mice bearing TC-1 tumors. Mice were either treated with a single intratumoral injection of NP(dox+pIC+R848+MIP3α) or a mock injection with PBS at day 8. The tumors and spleens were resected 10 days afterwards and analyzed ex vivo. Compared to the mock treated mice, the treated mice exhibited significantly higher levels of leukocytes in the tumor, as measured by cell staining for the pan-leukocyte marker CD45 (Figure 3A). Moreover, the treated mice showed significantly higher levels of CD3^+^ and CD4^+^ T cells in the tumor (Figures 3B & 3C). However, although they also showed higher levels of CD8^+^ T cells, this difference was not statistically significant (Figure 3D). In the spleen, the number of leukocytes was not found to differ significantly between the control and treated groups (data not shown). Moreover, no significant differences in the tumoral or splenic myeloid populations were observed between the two groups (Figures 3E & 3F). These results indicate that intratumoral treatment of TC-1 tumors with NP(dox+pIC+R848+MIP3α) enhances the lymphoid cell populations in the tumor but not in the spleen, and does not alter the myeloid population within the tumor microenvironment.

### Intratumoral co-delivery of dox and immune adjuvants by NPs augments the levels of circulating CD3^+^, CD8^+^ and cancer antigen-specific CD8^+^ T cells

**Figure 4:**
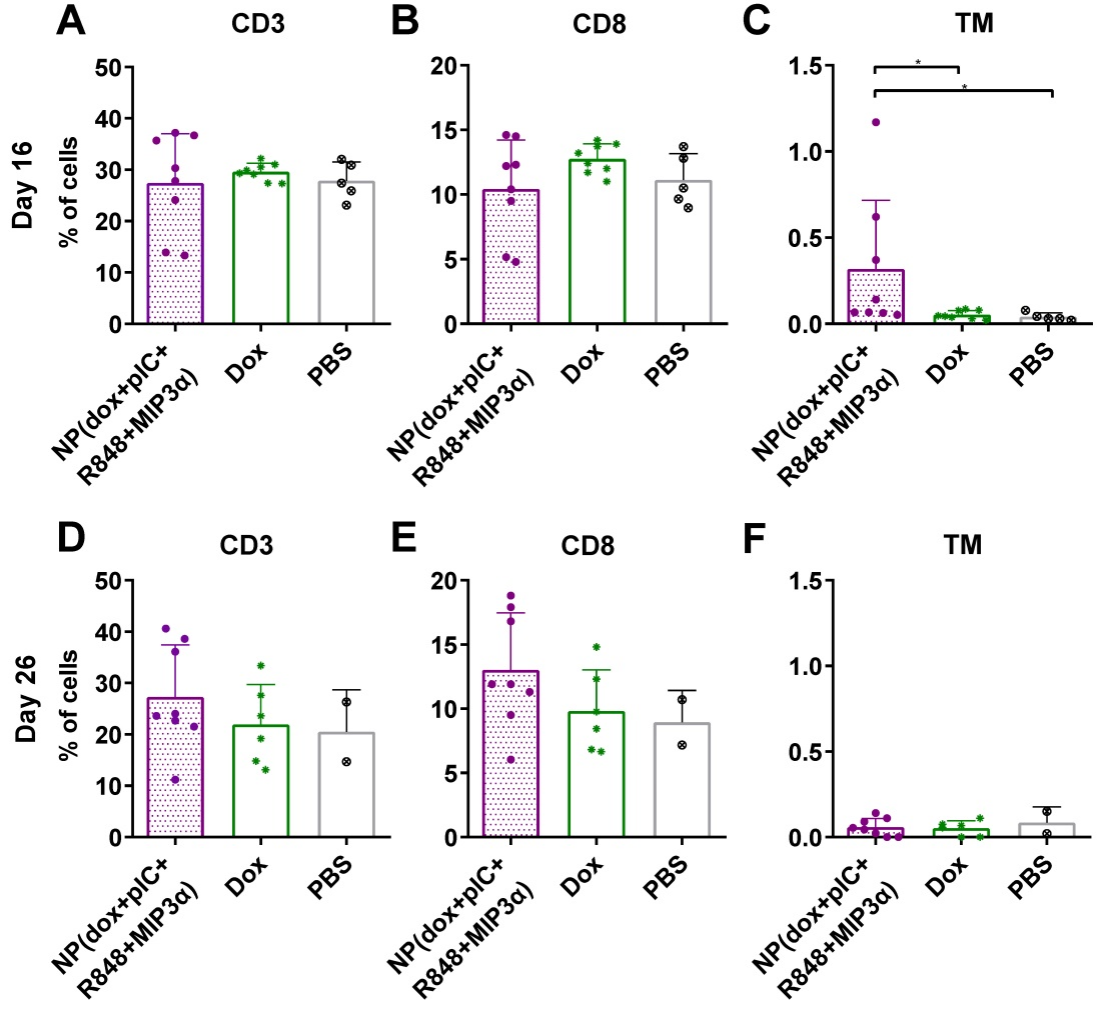
** Intratumoral co-delivery of dox and immune adjuvants by NPs augments the levels of circulating CD3^+^, CD8^+^ and cancer antigen-specific CD8^+^ T cells.** Quantification of CD3^+^, CD8^+^ and the HPV16 E7 tetramer specific T cells in blood at day 16 and at day 26 (8 and 16 days post-treatment) after treatment with intratumoral NP(dox+pIC+R848+MIP3α) as compared with free dox or PBS (mock control). **(A&B)** The levels of CD3^+^ and CD8^+^ T cells collected from blood of mice at day 16 (8 days after treatment) are depicted. n = 8 for NP(dox+pIC+R848+MIP3α), n = 8 for Dox and n = 5 for PBS. One representative out of two independent experiments. The differences between the groups are not statistically significant. **(C)** The levels of TM^+^ (cancer cell specific) CD3^+^CD8^+^ T cells collected from blood of mice at day 16 (8 days after treatment). n = 8 for NP(dox+pIC+R848+MIP3α), n = 8 for Dox and n = 5 for PBS. One representative out of two independent experiments. NP vs. dox (p=0.0351) and NP vs. PBS (p=0.0163). **(D&E)** The levels of CD3^+^ and CD8^+^ T cells collected from blood of mice at day 26 (18 days after treatment) are depicted. n = 8 for NP(dox+pIC+R848+MIP3α), n = 6 for Dox and n = 2 for PBS. One representative out of two independent experiments. The differences between the groups are not statistically significant. **(F)** The levels of TM^+^ (cancer cell specific) CD3^+^CD8^+^ T cells collected from blood of mice at day 26 (18 days after treatment). n = 8 for NP(dox+pIC+R848+MIP3α), n = 6 for Dox and n = 2 for PBS. One representative out of two independent experiments. The differences between the groups are not statistically significant. Statistics were calculated using a two-tailed Mann Whitney test. Statistical differences were considered significant at p < 0.05. * = p < 0.05; ** p = < 0.01; *** p < 0.001. All data are presented as mean ± SD. Abbreviations: TM: tetramer.

To determine whether the combined chemoimmunotherapy approach can alter the levels of circulating lymphocytes, we collected blood at day 16 and at day 26 (8 and 16 days post-treatment) from mice with TC-1 tumors and measured the number of CD3^+^, CD8^+^ and cancer antigen-specific CD8^+^ T cells. We observed that on day 16, the percentage of CD3^+^ and CD8^+^ T cells was not found to be significantly different (Figure 4A & 4B, respectively). However, treatment of mice with NP(dox+pIC+R848+MIP3α) induced a significant increase in cancer antigen-specific CD8^+^ T cells, compared to intratumoral administration of free dox or PBS alone (Figure 4C). At day 26, the average number of CD3^+^ and CD8^+^ T cells was higher in the blood of mice treated with NP(dox+pIC+R848+MIP3α) than mice treated with dox only, but this difference was not statistically significant (Figures 4D & 4E). In contrast to day 16, at day 26 there were no differences in the levels of cancer-specific CD8^+^ T cells among the three groups (Figure 4F).

### Intratumoral co-delivery of dox and immune adjuvants by NPs provides enhanced chemoimmunotherapeutic effects in mice with established tumors

**Figure 5:**
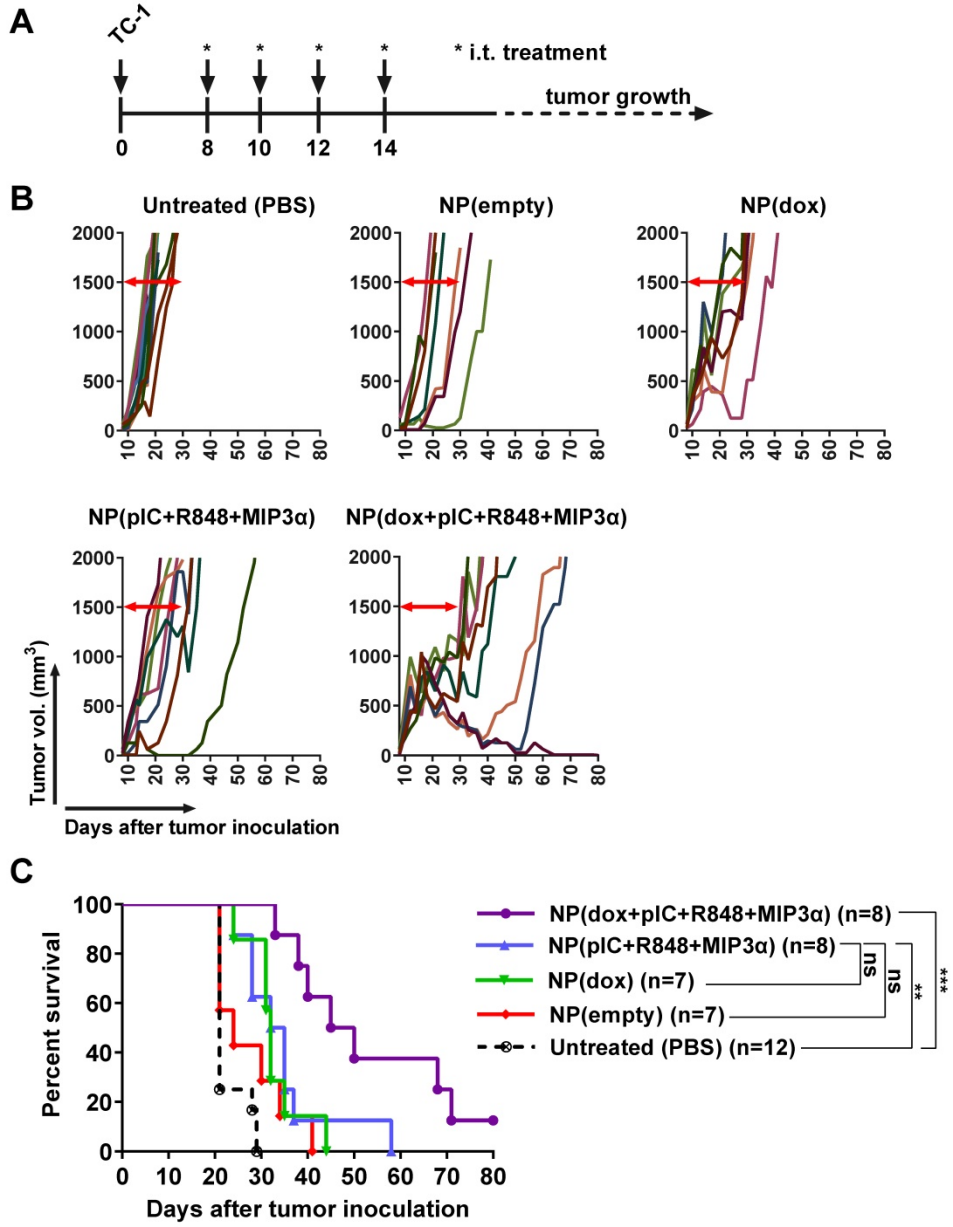
** Intratumoral co-delivery of dox and immune adjuvants by NPs provides enhanced chemoimmunotherapeutic effects in mice with established tumors. (A)** Schematic diagram of the TC-1 murine model experiment (C57BL/6 mice; n=8 per group, on average), showing inoculation and treatment days. **(B)** Tumor growth data from day 0 to day 80 for the PBS (control) group and four treatment groups (empty NPs, NP-delivered dox monotherapy, NP-delivered immune adjuvants and NP-delivered combination therapy). **(C)** Kaplan-Meier survival plots of pooled data, depicting progression-free survival and percent overall survival: NP(dox) vs. PBS p=0.0004; NP(pIC+R848+MIP3α) vs. PBS p=0.001; NP(dox+pIC+R848+MIP3α) vs. PBS p<0.0001; NP(dox+pIC+R848+MIP3α) vs. NP(pIC+R848+MIP3α) p=0.0082; NP(dox+pIC+R848+MIP3α) vs. NP(dox) p=0.0024; NP(empty) vs. PBS p=0.1082; NP(empty) vs. NP(dox) p=0.1160; NP(empty) vs. NP(pIC+R848+MIP3α) p=0.1076; NP(empty) vs. NP(dox+pIC+R848+MIP3α) p=0.0023. Survival curves were compared using the Gehan-Breslow-Wilcoxon test. Statistical differences were considered significant at * p = < 0.05; ** p = < 0.01; *** p < 0.001.

Next, we determined the respective therapeutic contributions of dox and of the immune adjuvants (pIC, R848 and MIP3α). Treatment was initiated with one intratumoral injection at 8 days post-inoculation, followed by three additional consecutive administrations at days 10, 12 and 14 (Figure 5A). The NPs were detectable with IVIS fluorescence imaging for at least 168 hours in the tumor after last injection (Figure S5). A significant therapeutic effect was observed for all the tumors treated with NPs containing dox alone, the immune adjuvants alone or the combination therapy but not for the empty NPs (Figures 5B & 5C). The greatest statistically significant therapeutic effect was provided by the combination therapy, followed by the monotherapies; however, there was no significant therapeutic difference between either monotherapy. These results corroborate an enhanced effect between dox and the immune adjuvants when intratumorally co-delivered by NPs.

### Intratumoral co-delivery of dox and immune adjuvants by NPs induces strong tumor regression and better overall survival than does of free components

**Figure 6:**
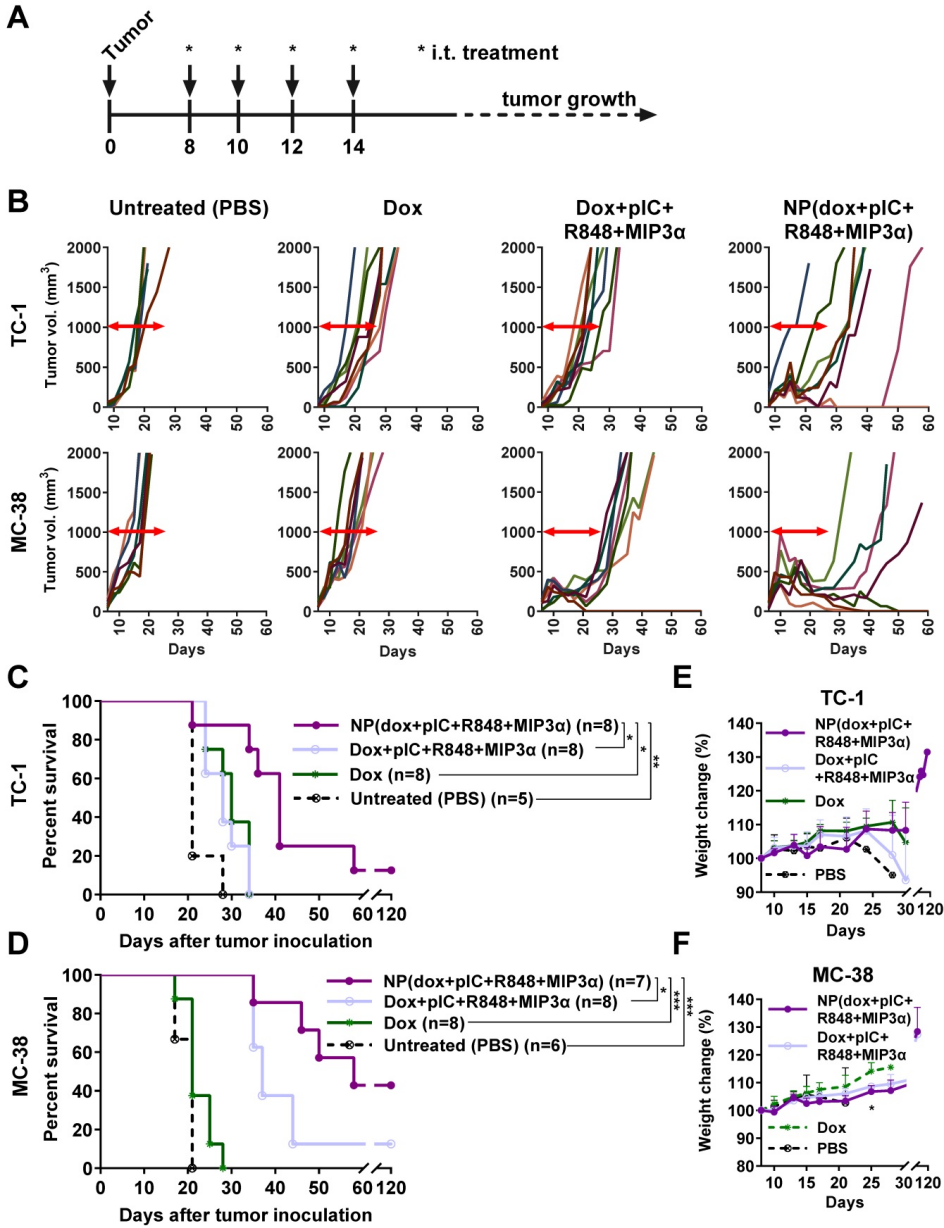
** Intratumoral co-delivery of dox and immune adjuvants by NPs induces strong tumor regression and better overall survival than does of free components. (A)** Schematic diagram of the TC-1 and MC-38 murine (C57BL/6 mice) model experiments, showing inoculation and treatment days. **(B)** Tumor-growth data from day 0 to day 60 for the PBS (control) group and three treatment groups (free dox, free combination therapy and NP-delivered combination therapy) in the TC-1 (top) and MC-38 (bottom) models. **(C)** Kaplan-Meier survival plots depicting progression-free survival and percent overall survival for the TC-1 model upon indicated treatments. n = 8 for each treatment group and n = 5 for PBS. NP(dox+pIC+R848+MIP3α) vs. PBS p=0.0041; Dox+pIC+R848+MIP3α vs. PBS p=0.0083; Dox vs. PBS p=0.0115; NP(dox+pIC+R848+MIP3α) vs. dox p=0.0113; NP(dox+pIC+R848+MIP3α) vs. Dox+pIC+R848+MIP3α p=0.0106. **(D)** Kaplan-Meier survival plots depicting progression-free survival and percent overall survival for the MC-38 model upon indicated treatments. n = 8 for NP(dox+pIC+R848+MIP3α), n=7 for Dox+pIC+R848+MIP3α, n=8 for Dox and n = 6 for PBS. NP(dox+pIC+R848+MIP3α) vs. PBS p=0.0008; Dox+pIC+R848+MIP3α vs. PBS p=0.0004; Dox vs. PBS p=0.1096; NP(dox+pIC+R848+MIP3α) vs. dox p=0.0004; NP(dox+pIC+R848+MIP3α) vs. Dox+pIC+R848+MIP3α p=0.0002. **(E)** The weight change of mice with TC-1 tumors after treatments. Data are presented as mean ± SD. **(F)** The weight change of mice with MC-38 tumors after treatments. Data are presented as mean ± SD. At day 25: NP(dox+pIC+R848+MIP3α) vs. dox p= 0.0121; Dox+pIC+R848+MIP3α vs. dox p= 0.0121. Survival curves were compared using the Gehan-Breslow-Wilcoxon test. Mice weight were analyzed by two-tailed Mann Whitney test. Statistical differences were considered significant at * p = < 0.05; ** p = < 0.01; *** p < 0.001.

To further assess the therapeutic advantage of our NPs, we compared intratumoral treatment of free dox, the free combination therapy (dox+pIC+R848+MIP3α) and the NP-delivered combination therapy in two murine models of cancer: MC-38 and TC-1, using immunocompetent C57BL/6 mice. Treatment was initiated at day 8, followed by three additional consecutive administrations at days 10, 12 and 14 (Figure 6A). The concentrations of the free compounds were matched to the concentrations of the compounds loaded inside the NPs. The tumors in mice treated with free dox monotherapy did not regress in either model (Figure 6B). Unlike the TC-1 tumors, the MC-38 tumors did initially respond to the free combination therapy. The greatest gain in overall survival in both models was observed for the NP-delivered combination therapy (Figure 6C & 6D). Importantly, halving the total dose of NP-delivered combination therapy and increasing the time between administrations gave sustained, measurable responses in both models, but failed to completely cure any mouse (Figures S6A to S6D). In both models, the effects of all treatments on weight gain was minimal (Figure 6E & 6F). However, at day 25, the weight of MC-38 mice treated with either combination therapy (NP or free) was slightly lower than that of the mice treated with dox alone. Furthermore, all the mice whose tumors had been eradicated later rejected a tumor re-challenge, which indicates development of functional immunological memory against tumor antigens (data not shown). In conclusion, these results indicate that the NP-delivered combination therapy of dox and immune adjuvants is more effective than the corresponding free therapy at inducing long-term tumor control and even complete remission in mice with MC-38 or TC-1 tumors and does not provoke any detectable side effects.

## Discussion

Here, we report that the NP mediated delivery of dox and immune adjuvants induces complete remissions and effective long-term tumor control in both lung and colon mice tumor models. We show that the combinatorial treatment of chemotherapy with non-specific immunotherapy induces superior therapeutic responses which are attained when biomaterial nanotechnology is employed for the co-delivery. Furthermore, we show that the NP mediated chemoimmunotherapy modality augments the levels of lymphocytes and of cancer specific CD8^+^ T cells in the tumor and circulating in blood, leading to tumor eradications.

For this paper, we prepared PEGylated PLGA NPs with an average size of approximately 180 nm, which is within the optimal functional range (40 nm to 300 nm) reported for drug-delivery NPs 32-34. When the NPs containing dox were co-cultured with cancer cells, more cancer cells were killed by dox inside NPs than an equal concentration of free dox. This finding could relate to a well-known drug efflux mechanism whereby transporters pump dox out of the cell 35. Indeed, NP-delivered drugs have been reported to bypass efflux transporters, which also corroborates our results 36. Nonetheless, the TC-1 cells were more resistant to dox treatment than the MC-38 cells, independently of the delivery method. We also analyzed the established tumors after treatment and within the cell marker panels tested, we did not find any significant changes within the myeloid populations. This could be due to tumor cells overcoming acute inflammatory cytokines triggered by the TLR agonists. However, we did observe significant increases in the numbers of lymphocytes in the tumor, but not in the spleen. Furthermore, we analyzed the blood of treated mice at two different time points and found that the combination therapy and the free dox monotherapy did not induce any reduction in the number of circulating lymphocytes. Together, these data indicate that, at the administered dose, the NP-delivered combination therapy did not reduce but rather increased the levels of lymphocytes in the tumor and did not affect the myeloid population within the parameters analyzed. However, at day 16 we found that only the combination treatment induced detectable numbers of cancer antigen-specific T cells. Similarly to radiotherapy or photo dynamic therapy, this evidences that cancer antigen-specific T cells can be generated without vaccination 37. Furthermore, we report that co-delivery of dox and the immune adjuvants in a single NP provided significantly longer progression-free survival and overall survival in treated mice bearing MC-38 or TC-1 tumors compared to untreated mice.

**Figure 7:**
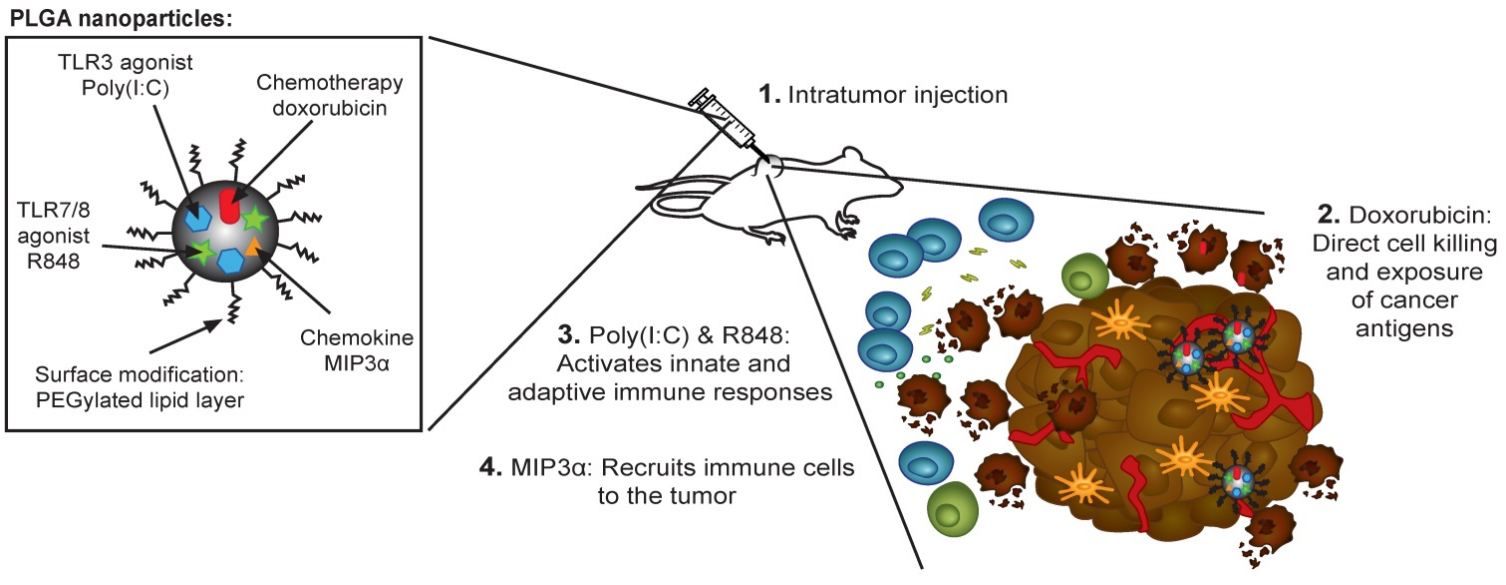
** Rational design of the nanoparticle-delivered chemoimmunotherapy to the tumor and tumor-draining lymph node. (Step 1)** The NPs are injected in the tumors, whereby a part of the NPs are endocytosed by cancer and cancer associated cells. The NPs that were not endocytosed start to release their content in the extracellular space of which a portion also drains to the tumor-draining lymph node (and further). Due to the good NP stability, the drug release and their biological effects is sustained for a prolonged period of time. **(Step 2)** The cytostatic doxorubicin induces (cancer) cell death and the release of cancer antigens. **(Step 3)** The immune modulators pIC and R848 activate residing immature and suppressed immune cells in the tumor and tumor-draining lymph node. **(Step 4)** MIP3α recruits more immune cells into the tumor.

Our NP-delivered combination therapy provides a triple mechanism based on the activity of dox, the chemokine MIP3α, and the TLR agonists pIC and R848 (Figure 7). Dox can induce the release of cancer antigens during cancer cell killing, but this effect alone often cannot provoke a sufficiently powerful immunological response for tumor clearance 38. The chemokine MIP3α, can amplify the intratumoral immune response by recruiting T cells to the tumor. Furthermore, given that our NP concomitantly delivers specific TLRs, their activity likely abrogates the immunosuppressive signals that tumor cells send to immature DCs that process tumor antigens. Specifically, as some of the TLR agonists that partially leak into blood stimulate dividing T cells, those remaining inside the tumor cells maintain a favorable T cell environment. Finally, while the PLGA NPs themselves are non-cytotoxic and biocompatible, the direct activation of the inflammasome by PLGA in DCs has been reported 39,40.

Our findings are consistent with those of other groups, who have reported the benefits of NPs for delivery of chemotherapy and non-specific innate immunotherapy 41-44. For instance, Roy et al. and Heo et al. treated murine B16 melanoma tumors with PLGA NPs containing paclitaxel and either a TLR4 or a TLR9 agonist, respectively 41,43. The authors observed an initial delay in tumor growth and a significant influx of lymphocytes into the tumors. Moreover, Yin et al. treated B16 tumors with PLGA NPs containing dox and interferon γ 44. The authors reported a delay in tumor growth, an influx of lymphocytes and NK cells into the tumors, and, in the tumor microenvironment, reduced levels of the suppressive cytokines IL-10 and TGFβ, and increased levels of IL-2 and TNFα.

Despite the promising results for NP-delivered combination therapies in animal models of cancer, the translation to clinical use must be judiciously guided. In the few clinical trials in which patients with solid tumors were treated TLR agonist monotherapies, the treatment caused some cancers to regress but caused others to proliferate and metastasize 45. For example, the strategy of activating TLR3 in lung cancer tumors appears to generate contradictory effects, inducing regressions in some tumors while conferring resistance in others 45,46. In contrast, colon cancer cells exposed to TLR3 agonists have been reported to initiate apoptosis more rapidly 45. The usage of slow-release vehicles, such as those enabled by nanotechnology, has been advocated for clinical therapy, since humans, unlike mice, are highly susceptible to cytokine release syndrome, a common side-effect of experimental immunotherapies 47-49.

Taken together, our results underscore the potential of NP-delivered chemoimmunotherapy to induce powerful anti-cancer immunity in solid, refractory tumors. We surmise that patients who are ineligible for surgery, or non-responsive to chemotherapy or immunotherapy, may benefit from this non-specific chemoimmunotherapy modality in the future.

## Figures and Tables

**Table 1:** Physicochemical characterization of the NPs

				Loading capacity (% w/w)				
Samples	Diameter	ζ Potential (mV)	PDI	NIR	Dox	pIC	R848	MIP3α
NP(NIR)-PEG Denoted as NP(empty)	187.4 ± 44.7	-13.9 ± 6.2	0.064	63.6 ± 1.4	-	-	-	-
NP(NIR+dox)-PEG Denoted as NP(dox)	185.9 ± 28.2	-13.5 ± 7.5	0.127	64.9 ± 0.9	13,9 ± 1.8	-	-	-
NP(NIR+pIC+R848+MIP3α)-PEG Denoted as NP(pIC+R848+MIP3α)	177.3 ± 86.6	-14.3 ± 4.9	0.120	61.1 ± 7.8	-	47.7 ± 2.6	58.4 ± 3.2	63.8 ± 5.0
NP(NIR+dox+pIC+R848+MIP3α)-PEG Denoted as NP(dox+pIC+R848+MIP3α)	177.3 ± 86.8	-14.3 ± 4.9	0.120	62.8 ± 5.6	6.3 ± 1.1	37.9 ± 10.1	17.1 ± 3.8	63.9 ± 3.8

Physicochemical characterization of the PLGA-PEG NPs containing dox and/or different immune adjuvants. The PLGA NPs were characterized by dynamic light scattering and zeta potential measurements. PLGA NPs size and zeta potential data represent the mean value ± SD of 10 readings of one representative batch. The loading capacity of dox and NIR dye was measured by fluorescence method. The loading capacity of pIC, R848 and MIP3α was determined by RP-HPLC analysis. The loading capacity data represent the average value ± SD of batch variation.

## Abbreviations

AFM: Atomic Force Microscopy
Dox: doxorubicin
DCs: dendritic cells
IFNs: type I interferons
MIP3α: Macrophage Inflammatory Protein-3 alpha
NIR: Near infrared
NP: nanoparticle
pIC: Poly(I:C)
PLGA: poly(lactic-co-glycolic acid)
TEM: Transmission Electron Microscope
TLR: Toll-like receptor
TM: tetramer
