# Supplementary material for: Effective chemoimmunotherapy by co-delivery of doxorubicin and immune adjuvants in biodegradable nanoparticles
Source: Theranostics. 2019 Aug 21;9(22):6485–500. doi: 10.7150/thno.34429 (PMC6771237; doi:10.7150/thno.34429)
Supplement: Supplementary file 1 — Supplementary figures. [file thnov09p6485s1.pdf]

Effective chemoimmunotherapy by co-delivery of doxorubicin and immune adjuvants in biodegradable nanoparticles

Candido G. Da Silva, Marcel G.M. Camps, Tracy M.W.Y. Li, Luana Zerrillo, Clemens W. Löwik, Ferry Ossendorp, Luis J. Cruz

Supplementary Figures

Figure S1. Flow cytometry gating strategy
Figure S2. The size and zeta potential data characterized by dynamic light scattering
Figure S3. Stability study of PLGA NPs
Figure S4. Cytotoxicity of the drug-loaded NPs vs. solvent controls
Figure S5. IVIS imaging of TC-1 tumors after treatment
Figure S6. Halving the dose of NP-delivered combination therapy does not alter its anti-tumor efficacy but does lead to lower overall survival

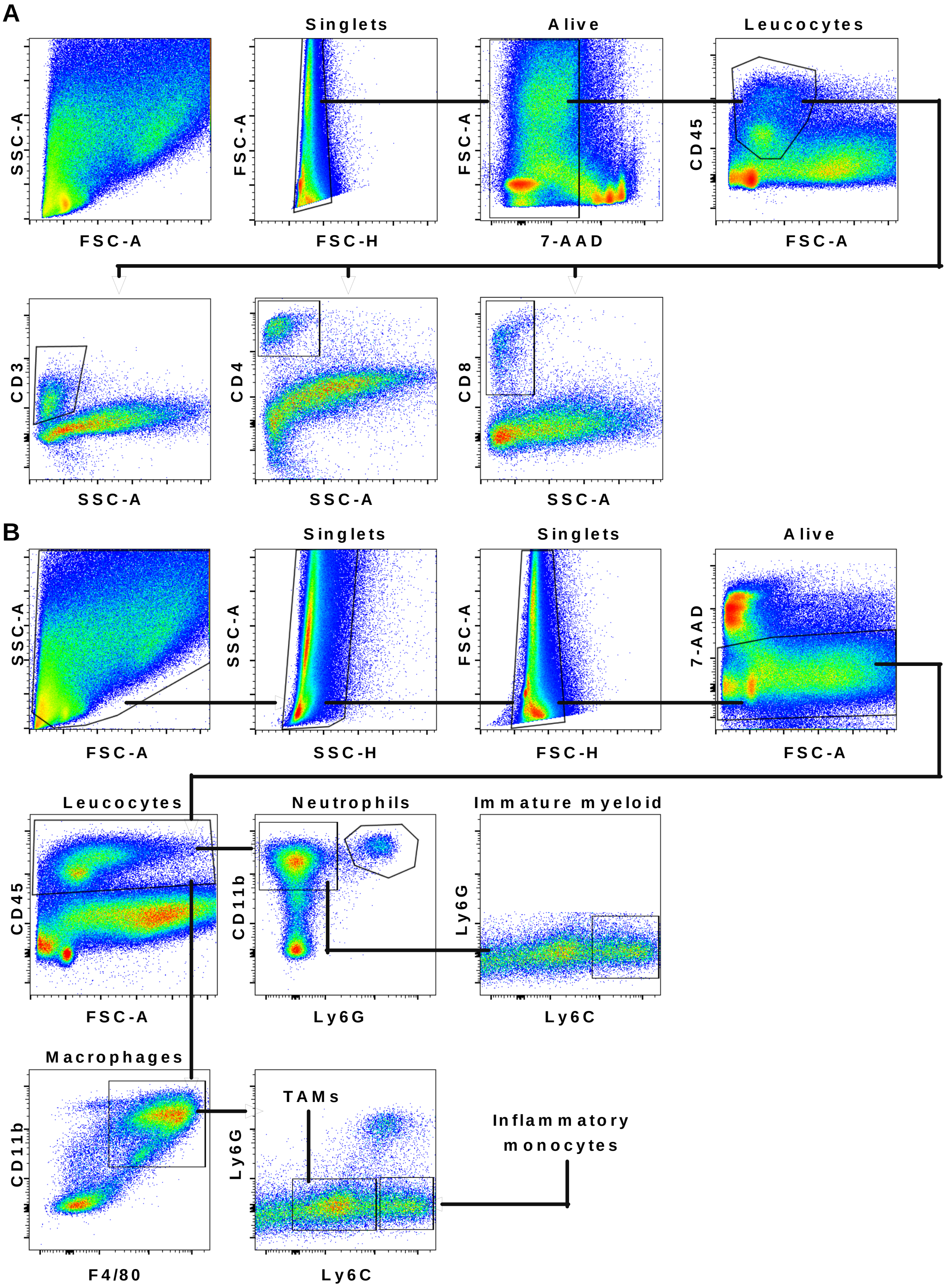

Figure S1. Flow cytometry gating strategy

A) Flow cytometry gating strategy for the lymphoid populations. **B)** Flow cytometry gating strategy for the myeloid populations.

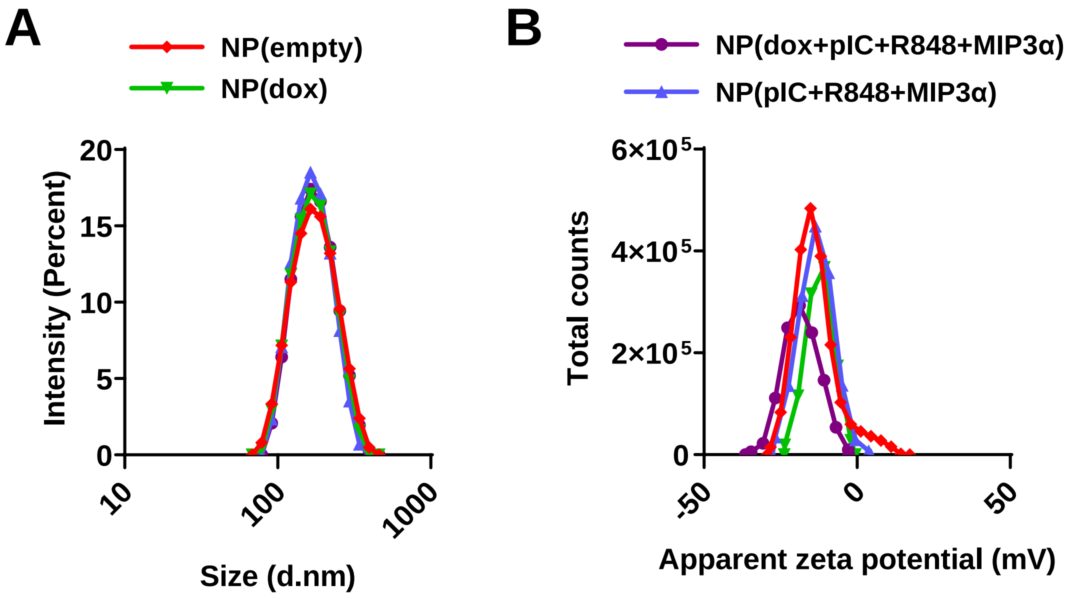

Figure S2. The size and zeta potential data characterized by dynamic light scattering

The size (**A**) and zeta potential (**B**) data distributions represent the mean value ± SD of 10 readings.

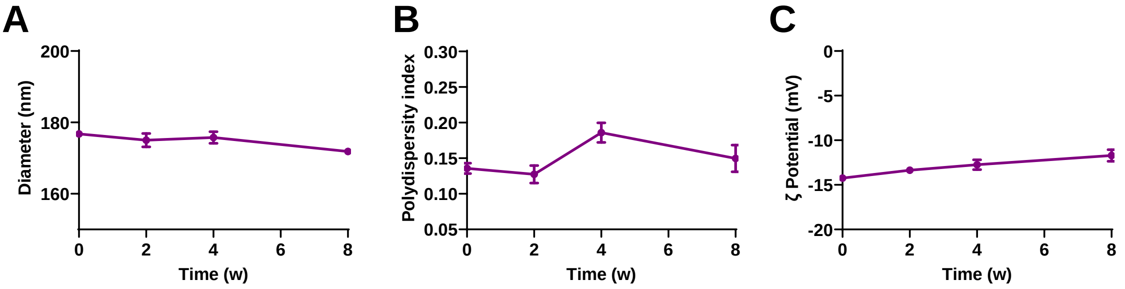

Figure S3. Stability study of PLGA NPs

NPs were incubated in PBS at room temperature and at constant rotation movement. Samples were taken at described time points and characterized by dynamic light scattering and zeta potential measurements. **A)** NP size stability study. **B)** NP polydispersity index (PDI) stability study. **C)** NP ζ potential stability study. n = 3 from one representative experiment from a representative NP batch. Data are presented as mean ± SEM.

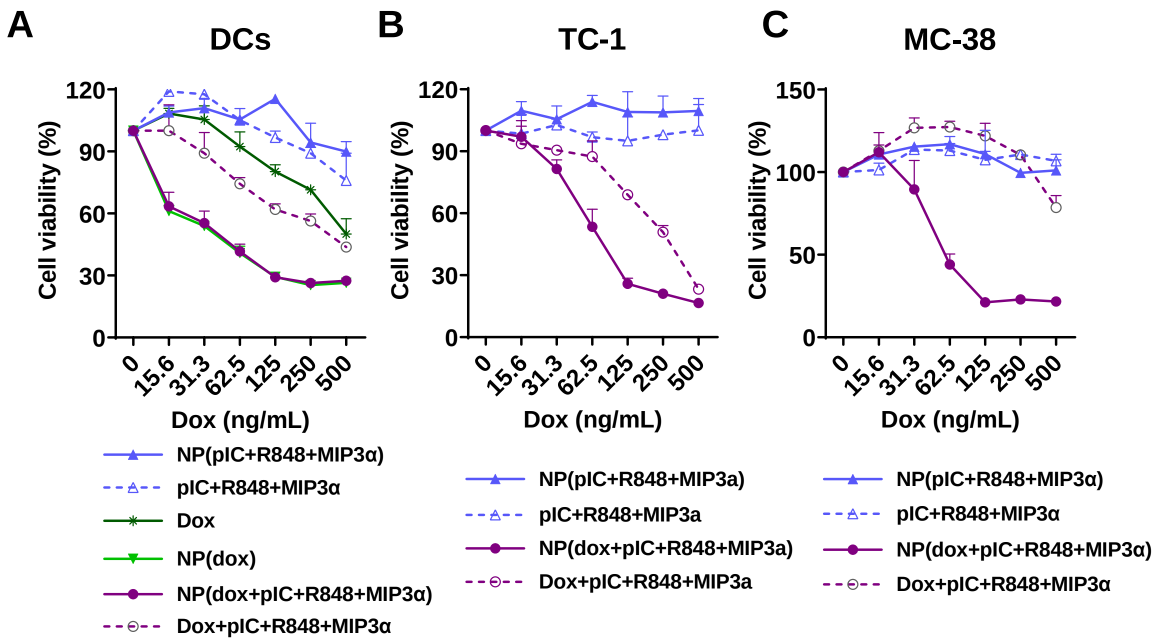

Figure S4. Cytotoxicity of the drug-loaded NPs vs. solvent controls

Cell viability assessed by MTS cell proliferation assay upon 72 hours incubation with indicated compounds on DCs (A), TC-1 (B) or MC-38 (C) cells. n = 3 from one representative out of two independent experiments. All data are presented as mean ± SD.

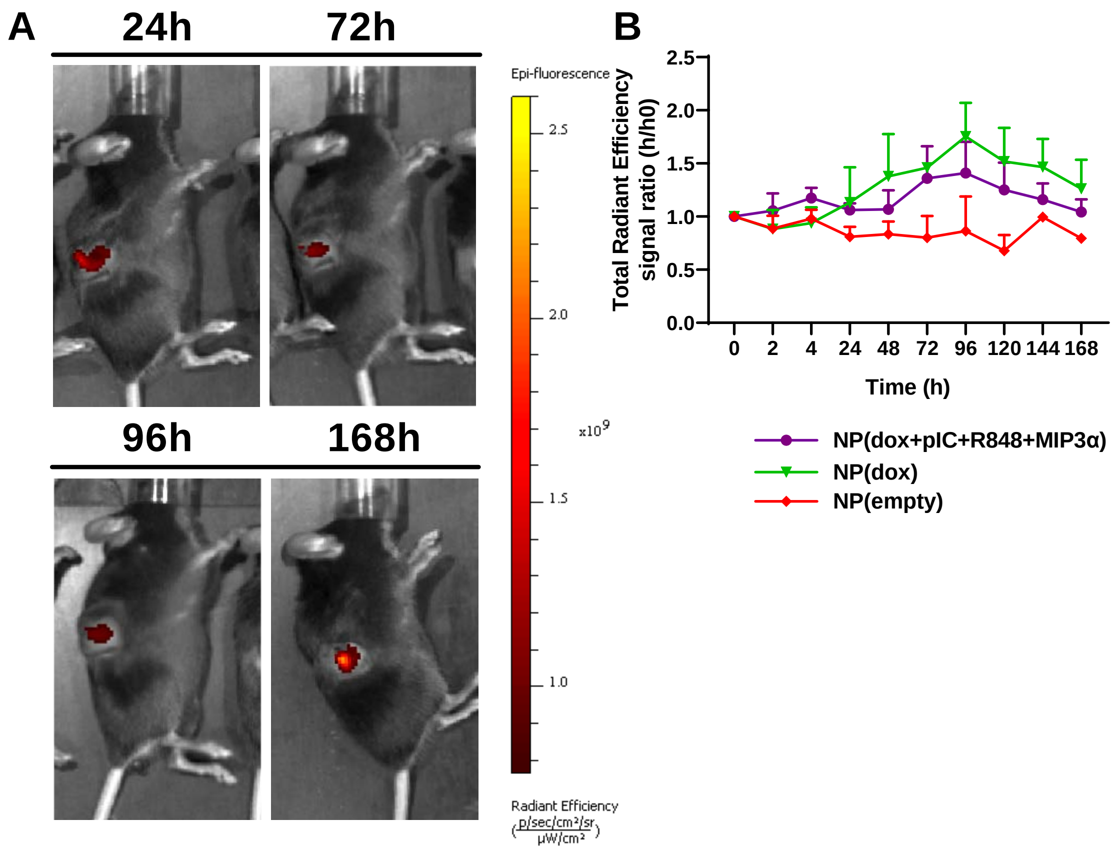

Figure S5. IVIS imaging of TC-1 tumors after treatment

A) Representative IVIS image of a mice with a TC-1 tumor in the flank followed from 24 to 168 hours after last injection with NP(dox+pIC+R848+MIP3a). **B)** Graph shows the quantification of the total radiant efficiency ([p/s]/[µW/cm²]) signal ratio (h/h0) in tumors injected with indicated NPs over time. n = 5 from one representative experiment. Data are presented as mean ± SEM.

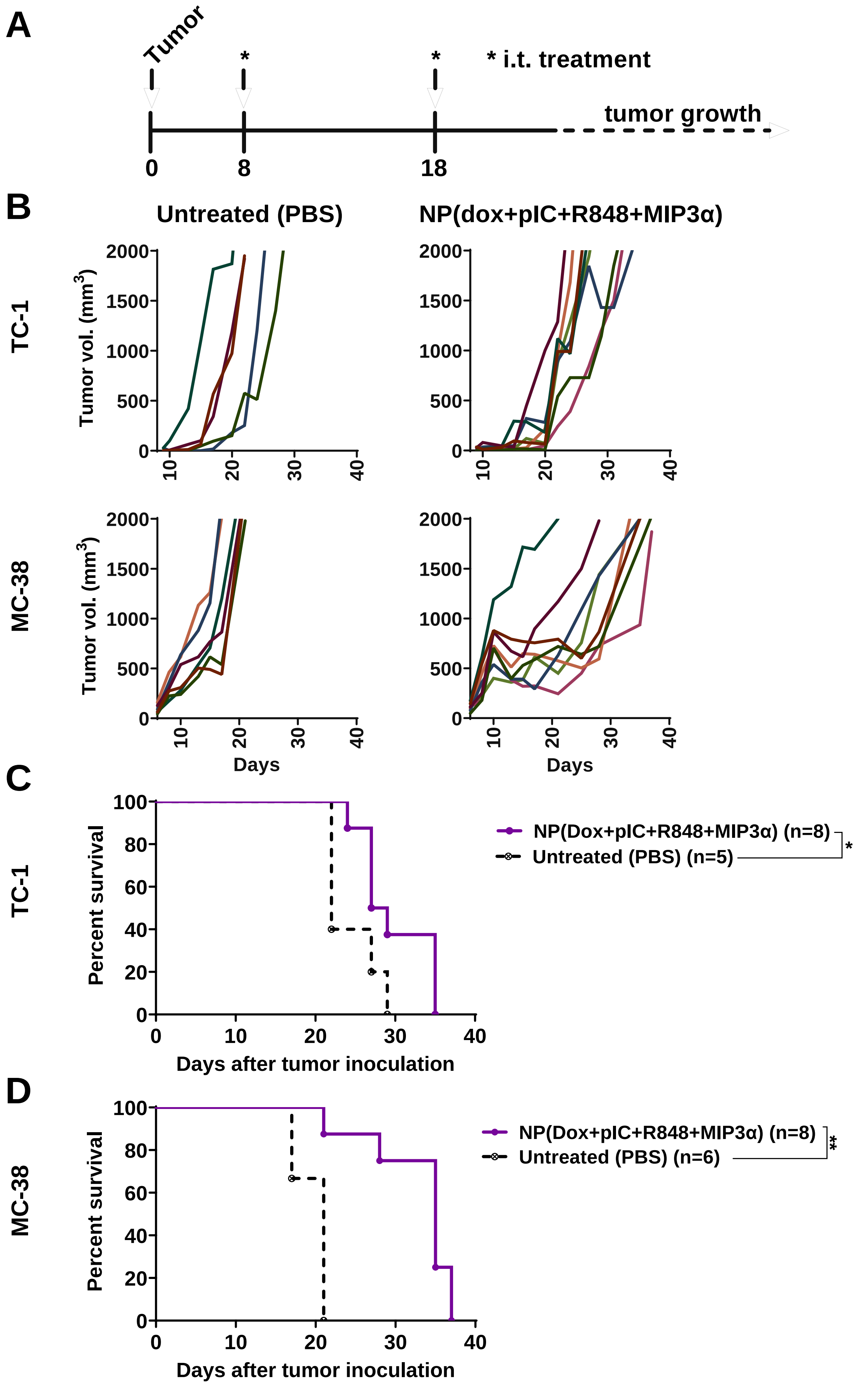
A
Tumor
* * * i.t. treatment
tumor growth
0 8 18
B
Untreated (PBS)
NP(dox+pIC+R848+MIP3α)
TC-1
Tumor vol. (mm3)
MC-38
Days
C
TC-1
Percent survival
Days after tumor inoculation
NP(Dox+pIC+R848+MIP3α) (n=8)
Untreated (PBS) (n=5)
*
D
MC-38
Percent survival
Days after tumor inoculation
NP(Dox+pIC+R848+MIP3α) (n=8)
Untreated (PBS) (n=6)
**

Figure S6. Halving the dose of NP-delivered combination therapy does not alter its anti-tumor efficacy but does lead to lower overall survival

A) Schematic diagram of the TC-1 and MC-38 murine (C57BL/6 mice) model experiments, showing inoculation and treatment days (n=8 mice per group, on average). **B)** Tumor growth data from day 0 to day 40 for the PBS (control) group and NP-delivered combination therapy group in the TC-1 (top) and MC-38 and (bottom) models. **C)** Kaplan-Meier survival plots depicting the length of progression-free survival and the overall survival (as %) for the TC-1 model: NP(dox+pIC+R848+MIP3α) vs. PBS p=0.038. **D)** Kaplan-Meier survival plots depicting the length of progression-free survival and the overall survival (as %) for the MC-38 model: NP(dox+pIC+R848+MIP3α) vs. PBS p=0.0014. Survival curves were compared using the Gehan-Breslow-Wilcoxon test. Statistical differences were considered significant at * p = < 0.05; ** p = < 0.01; *** p < 0.001.
